# Review of The Physics and Technology of Radiation Therapy, 2^nd^ ed., by McDermott and Orton

**DOI:** 10.1002/acm2.12737

**Published:** 2019-09-26

**Authors:** Joseph P. Driewer

**Affiliations:** ^1^ Nebraska Methodist Health System in Omaha Omaha NE USA

## 1 OVERVIEW

The 2^nd^ edition of The Physics and Technology of Radiation Therapy aims to provide a comprehensive treatment of medical radiation therapy physics for therapists, dosimetrists, and physician residents. Book chapters cover the range of radiation physics material that is found in certification exams in those areas. These exam topics are helpfully identified and cross‐referenced in outline form in Appendix A, which makes this text a valuable and recommended study resource for students and residents.

## 2 GOALS OF THE 2ND EDITION

This second edition work, published in 2018, seeks to update a well‐received 2010 book in medical radiation therapy physics. In the first edition, the authors sought to fill a void in physics education. That is, while there were good textbook options for physics students and other advanced students in the area, nothing was written to meet directly the educational needs of therapy, dosimetry, and physician members of the radiation team. This emphasis of the text has not changed in the second edition.

What has changed are several aspects of the content to bring the textbook up‐to‐date. The authors have added short sections on digital imaging and OLSDs, for instance. Even more substantive changes between the first and second editions are the inclusion of IMRT and proton therapy as separate chapters, as well as the reworking of several chapters to remove discussions of technologies such as Co‐60 teletherapy and simulators.

By and large, the authors have met their goal of bringing the text up‐to‐date on technology. To be sure, it’s impossible to include every technological advancement in radiation therapy over the last 10 years in a new book. But that is at best a secondary goal of this work (acknowledged in the preface). Primarily, the authors sought to protect the educational value of the text. Chapter summaries, rules of thumb for calculations, numerous worked problems, question sets, and call‐out boxes all demonstrate that this is a text to be interacted with, not merely consumed. In that the text is successful.

## 3 ORGANIZATION AND FEATURES

The book contains 22 chapters, including two chapters of math and general physics review, and four appendices. The chapters do build nicely in the current order, although some could stand on their own. At a high level, the chapters progress through radiation production, interactions, quantities, and measurement, and then move to various applications, such as medical accelerators, calibrations, and descriptions of and applications of dose in patients. Advanced therapy modalities are covered in later chapters. Helpfully, each chapter contains a brief bulleted summary of important concepts and definitions (except the first two chapters of review). Appendix A contains outlines of certification exams that are cross referenced to textbook sections for ease of use. Students can very easily look through these outlines for areas in need of review and flip to that section of the text. Based on these outlines, the MDCB and AART exams, as well as core medical physics topics for radiation oncology resident training, are covered well in this book. Appendix B and C contain beam data of a fictitious medical linac, the Mevelac, for working sample problems. Appendix D contains answers to selected problems.

As a user of the first edition textbook in a classroom setting, I would have rather had traditional page numbers than section‐page format that does remain unchanged in the second edition. One obvious change, however, is that this text is printed in color. The color palate in the first edition made for awkward page flipping, but this problem has been corrected as color figures are included throughout the text. The figures themselves remain largely unchanged. Rules of thumb, worked sample problems, and important tables are highlighted in consistent colors throughout the text which does draw attention to them.

## 4 CHAPTER CONTENTS

By and large, chapters on x ray production, radioactivity, radiation interactions, radiation quantities, detection, MU calculations, brachytherapy, and radiation protection are unchanged and are well‐covered for the target audience. Additionally, the text is not ostentatious at all, which in my experience does facilitate use of the book. Anecdotally, when my group decided to switch to the first edition of this text for a therapy physics class, most evaluation comments were positive. This seemed to be a book that students would actually read! I suspect that the second edition will also hold up in that regard.

There is still broad coverage of therapy physics topics. For this reason, instructors will still likely have to decide what is best for their own class. For example, TG‐43 is covered but perhaps this topic would only be relevant to residents. Outside of the target audience, medical physics residents may find the summaries helpful, but should look for more detailed information elsewhere, for example on cavity theory, optimization, and Monte Carlo.

## 5 SUMMARY OF ASSESSMENT

The 2nd edition of The Physics and Technology of Radiation Therapy by McDermott and Orton is an accessible textbook for radiation therapists, medical dosimetrists, and radiation oncology residents. It could be a great primary text for a first course in radiation therapy physics as well as a great study resource for board exams in these areas.

## 6 ABOUT THE ATHUOR

Joseph Driewer, Ph.D., DABR, is Chief Physicist for Nebraska Methodist Health System in Omaha, NE.

